# ﻿A new species of *Mollinedia* (Monimiaceae, Laurales) from the Quadrilátero Ferrífero, Brazil

**DOI:** 10.3897/phytokeys.234.109804

**Published:** 2023-10-23

**Authors:** Danilo Alvarenga Zavatin, Renato Ramos, Mauricio Takashi Coutinho Watanabe, Luciano Gonçalves Pedrosa, Elton John de Lírio

**Affiliations:** 1 Universidade de São Paulo, Instituto de Biociências, Rua do Matão 277, Edifício Sobre-as-ondas (Herbário), São Paulo, SP, 05508-900, Brazil Universidade de São Paulo São Paulo Brazil; 2 Instituto Tecnológico Vale, Rua Boaventura da Silva 955, Belém, PA, 66055-090, Brazil Instituto Tecnológico Vale Belém Brazil; 3 Departamento de Biodiversidade, Evolução e Meio Ambiente, Universidade Federal de Ouro Preto, Campus Morro do Cruzeiro, ICEB III, Ouro Preto, MG, 35400-000, Brazil Universidade Federal de Ouro Preto Ouro Preto Brazil

**Keywords:** Flora of Brazil, Magnoliidae, Mollinedieae, new taxon, Flora do Brasil, Magnoliidae, Mollinedieae, novo taxon

## Abstract

Monimiaceae comprises approximately 26 genera and 250 species, with a pantropical distribution, predominantly occurring in humid forests. In Brazil, it is represented by five genera and 47 species, most of which are found in the Atlantic Forest, particularly in dense ombrophilous forest. Nevertheless, studies on this family in other biomes and vegetation types in Brazil are still scarce. The Quadrilátero Ferrífero (QF), a region located in the state of Minas Gerais, exhibits high plant species richness and endemism. During collections and analysis of herbarium materials from this region, some specimens of *Mollinedia* caught our attention due to a combination of characteristics that do not match those of any described species within the genus. For this reason, we describe this new taxon and assess its risk of extinction. Additionally, we review the occurrences of Monimiaceae in the QF and provide maps of its geographical distribution. With the description of this new species, the region now hosts eight species of Monimiaceae, two from the genus *Macropeplus* and six from *Mollinedia*. The new species is the first endemic species of the Monimiaceae family to be described in the QF. Concerning the extinction risk assessment, the new species was assessed as critically endangered.

## Abstract (es)

Monimiaceae abrange c. 26 gêneros e 250 espécies, tem distribuição pantropical e suas espécies ocorrem predominantemente em florestas úmidas. No Brasil está representada por cinco gêneros e 47 espécies, a maior parte das quais com ocorrência na Mata Atlântica, em floresta ombrófila densa. Estudos com a família em outros biomas e vegetações ainda são escassos no Brasil. O Quadrilátero Ferrífero (QF), região localizada no estado de Minas Gerais, possui alta riqueza e endemismo de plantas. Durante coletas e análise de materiais herborizados da região, alguns espécimes de *Mollinedia* nos chamaram a atenção devido a uma combinação de caracteres que não coincidem com nenhuma espécie descrita para o gênero. Este novo táxon é aqui descrito e tem seu risco de extinção avaliado, além disso, revisamos as ocorrências de Monimiaceae no QF, e fornecemos mapas de distribuição geográfica. Com a descrição desta nova espécie, a região apresenta agora oito espécies de Monimiaceae, duas do gênero *Macropeplus* e seis de *Mollinedia*. A nova espécie é a primeira da família endêmica do QF. Em relação à avaliação do risco de extinção, a nova espécie foi categorizada como Criticamente em Perigo.

## ﻿Introduction

Monimiaceae is a pantropical family of shrubs, trees or lianescent plants with opposite leaves showing acuminate-convex teeth on the margin and venation entering the tooth medially and not joined by lateral veins, called “monimioid teeth” (Hickey and Wolf 1975). The family has 26 genera and c. 250 spp. occurring mainly in humid forests (Philipson 1993; Renner et al. 2010; Lírio et al. 2020a). On the American continent, ca. 60 species occur in six genera: *Grazielanthus* Peixoto & Per.-Moura, *Hennecartia* J. Poiss., *Macrotorus* Perkins, *Macropeplus* Perkins, *Mollinedia* Ruiz and Pavón and *Peumus* Molina (Lorence 1999; Lírio et al. 2020a, b). *Mollinedia* is the richest Neotropical genus of the family, with c. 45 species, occurring in southern Mexico and Central and South America (Lírio et al. 2015, 2020a). *Mollinedia* is characterized by staminate flowers with nearly rounded buds, tepals with a ratio of ca. 1:1 in relation to the length of the flower, ovate or rounded stamens, and locules with two longitudinal openings and an extended connection or confluence at the apex, making the anther horseshoe-shaped (Perkins 1898, 1900; Lírio et al. 2020a). However, phylogenetic analyses suggest that this morphologically well-circumscribed genus is not monophyletic, albeit with low statistical support (Renner et al. 2010). In Brazil, there are 38 species of *Mollinedia*, 33 of which occur in the Atlantic forest, four in the Amazon forest, three in the Cerrado, and one in Pantanal (Lírio et al. 2020a). To date, 28 species of *Mollinedia* have been officially assessed at the national level following IUCN criteria, with 11 of them being considered threatened with extinction and one as deficient data (Lírio et al. 2023a). In addition, many *Mollinedia* species are microendemic, known by few collections in herbaria and/or considered rare (Lírio et al. 2018, 2023a).

The “Quadrilátero Ferrífero” (QF) region belongs to Minas Gerais state (Brazil) and is situated in a transition zone between the Atlantic Forest and Cerrado domains. The total area is approximately 7,200 km^2^ and has significant iron ore reserves (Jacobi et al. 2007; Messias et al. 2012). At the highest altitudes, ironstone outcrop vegetation (named “canga”) can be found. This ecosystem is considered a center of high diversity, including the occurrence of poorly known, endemic and endangered plant and animal species (Jacobi et al. 2007; Zappi et al. 2017).

**Figure 1.**
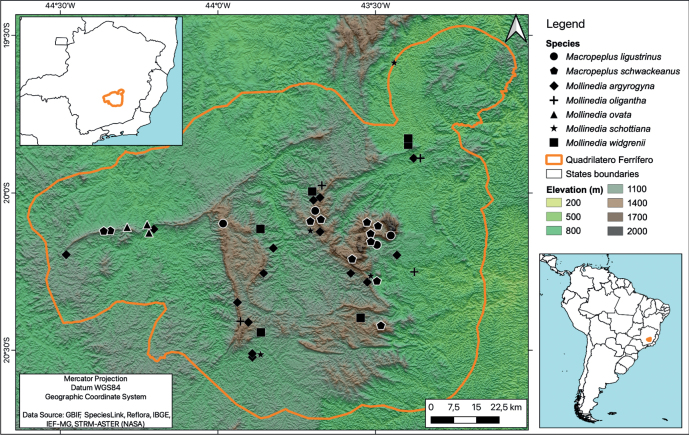
Geographic distribution map of Monimiaceae species in the Quadrilátero Ferrífero, state of Minas Gerais, Brazil, showing the limits of the region, mountainous areas and elevations.

Seven species of Monimiaceae have been registered in the QF region, none of which are restricted to this area, five of which belong to *Mollinedia* and two of which belong to *Macropeplus* (Fig. 1). Here, a new species of *Mollinedia* endemic to the QF is described and illustrated. Additionally, its conservation status is evaluated, and its morphological affinities are discussed.

## ﻿Material and methods

The newly described species was recognized by a unique combination of features (Donoghue 1985) identified through comparisons with morphologically similar taxa and a literature review (Peixoto 2002; Lírio and Peixoto 2017; Lírio et al. 2020a). A 10–60× magnification stereomicroscope was used to analyze the morphological features of the specimens. Terminology follows that of Harris and Harris (2001) for general morphology, except for characters unique to Monimiaceae, which are described according to Hickey (1973), Hickey and Wolfe (1975) and Perkins (1898, 1900). Herbarium acronyms follow Thiers (2023, continuously updated). The comparison with similar species was conducted through analysis of collections deposited in the following herbaria: BHCB, BHZB, GH, HAS*, ICN*, INPA, LZ*, MA, MBM, MBML, MO*, NY*, OUPR, SP, SPF, P, RB and W. Specimens from collections indicated with “*” were studied based on digital images available in virtual herbaria (REFLORA 2023; SpeciesLink 2023). IUCN (2012, 2022) conservation status was applied to assess and analyze the threat criteria at the interface with the anthropogenic factors fire (INPE 2023) and land natural or anthropogenic cover (IDE-SISEMA 2019). Species area values were determined using GEOCAT (Bachman et al. 2011). Environmental characteristics were mapped using soil data (UFV 2010a, b), elevation data (NASA et al. 2019) and 10-meter-resolution normalized difference vegetation index (NDVI) data from the Sentinel-2 databases processed in the *rgee* package (Aybar et al. 2020) of R (R core Team 2018). A distribution map was produced in QGIS version 3.16 (QGIS Development Team 2021).

## ﻿Taxonomic treatment

### ﻿ Mollinedia fatimae

Taxon classificationPlantaeLauralesMonimiaceae

Zavatin & Lírio sp. nov.

972CCDA4-3452-5BF3-B8FC-CBBF1F6A5BF6

urn:lsid:ipni.org:names:77329058-1

Figs 2 , 3 , 4

#### Type.

Brazil, Minas Gerais: Ouro Preto, Parque Estadual do Itacolomi, mata em frente à capela, 20°26'04"S, 43°30'37"W, 1343 m elev., 28 Nov 2022, mal. fl. *D.A. Zavatin & L.G. Pedrosa 1327* (holotype: SPF!, isotypes (to be distributed to): ALCB!, B!, BHCB!, BHZB!, DIAM!, HUFU!, HURB!, K!, MEXU!, MO!, NY!, OUPR!, P!, PTBR!, RB!, UB!).

#### Diagnosis.

*Mollinediafatimae* resembles *Mollinediaboracensis* due to its coriaceous leaf consistency; however, it can be easily differentiated by the leaves glabrate on the abaxial surface (vs. glabrous), flowers farinose-pilose (vs. puberulous), stamens 14–20 (vs. 22–24) and carpels 12–20 (vs. 26–30).

***Trees***, ca. 6−15 m tall, dioecious; rhytidome rough, twigs cylindrical, young branches strigulose, covered by whitish and long simple trichomes; older branches reddish, glabrescent, with conspicuous lenticels. ***Leaves*** 5.5−12.5 × 1.5−2.8 cm, opposite, narrowly elliptic, apex attenuate or usually rounded, base attenuate, margin entire or with 1−5 teeth per side, not in pairs, irregularly distributed between sides, from the upper half to the apex, coriaceous, discolored, brown when dried, lighter on the abaxial surface, young leaves white-strigose on both surfaces; denser on primary vein of the abaxial surface, then glabrate on the abaxial surface and glabrous on the adaxial surface, primary vein apparent on the adaxial surface and prominent on the abaxial surface, whitish pubescence adpressed on the abaxial surface, mostly along the basis of the primary vein, secondary veins 4−6 pairs, not apparent or only slightly apparent on the adaxial surface and prominent on the abaxial surface, petiole 0.5−1.1 cm long, canaliculated, puberulous or glabrous on older leaves. Inflorescences in thyrses or fascicles with up to 3 cymes (3−florous), axillary or terminal. ***Staminate flowers*** greenish, 5 × 5 mm, indumentum of two types, farinose and pilose, caducous on anthesis, rachis 1.5−3 cm, peduncle 3−8 mm, pedicel 4−8 mm, bracts ovate, apex acute or rounded, c. 1 mm long, bracteoles lanceolate, apex acute, 2−3 mm long, whitish tomentose or when absent, with a ferruginous scar, receptacle campanulate, tepals c. ¾ of the flowers length, externals ovate, apex obtuse, internals ovate, apex truncate or acuminate, stamens, 14−20, ovate, locules confluent at the apex, filament short. ***Pistillate flowers*** greenish, 5 × 6 mm, fascicules up to 2 flowers, rarely solitary, indumentum of two types, farinose and pilose, peduncle 0.4−2 cm, pedicel 1−1.7 cm, bracts ovate, apex acute or rounded, c. 1 mm long, bracteoles lanceolate, apex acute, 2−3 mm long, whitish tomentose or when absent, with a ferruginous scar, receptacle cupuliform, internally puberulous, tepals c. ¾ of the flower length, externals ovate with a caudate apex, margin entire, internals ovate with truncate or acuminate apex, carpels 12−20, ovary oblong, stigma c. 1/3 of the carpel length. ***Drupelets*** ellipsoid, 0.8−1.5 × 0.7−1.3 cm, not stipitate, apex rounded, stigma persistent, brown when dried, fruiting receptacle 0.6−1 cm wide, reflexed, fruit scars prominent, peduncle plus pedicel 1−1.7 cm long.

**Figure 2.**
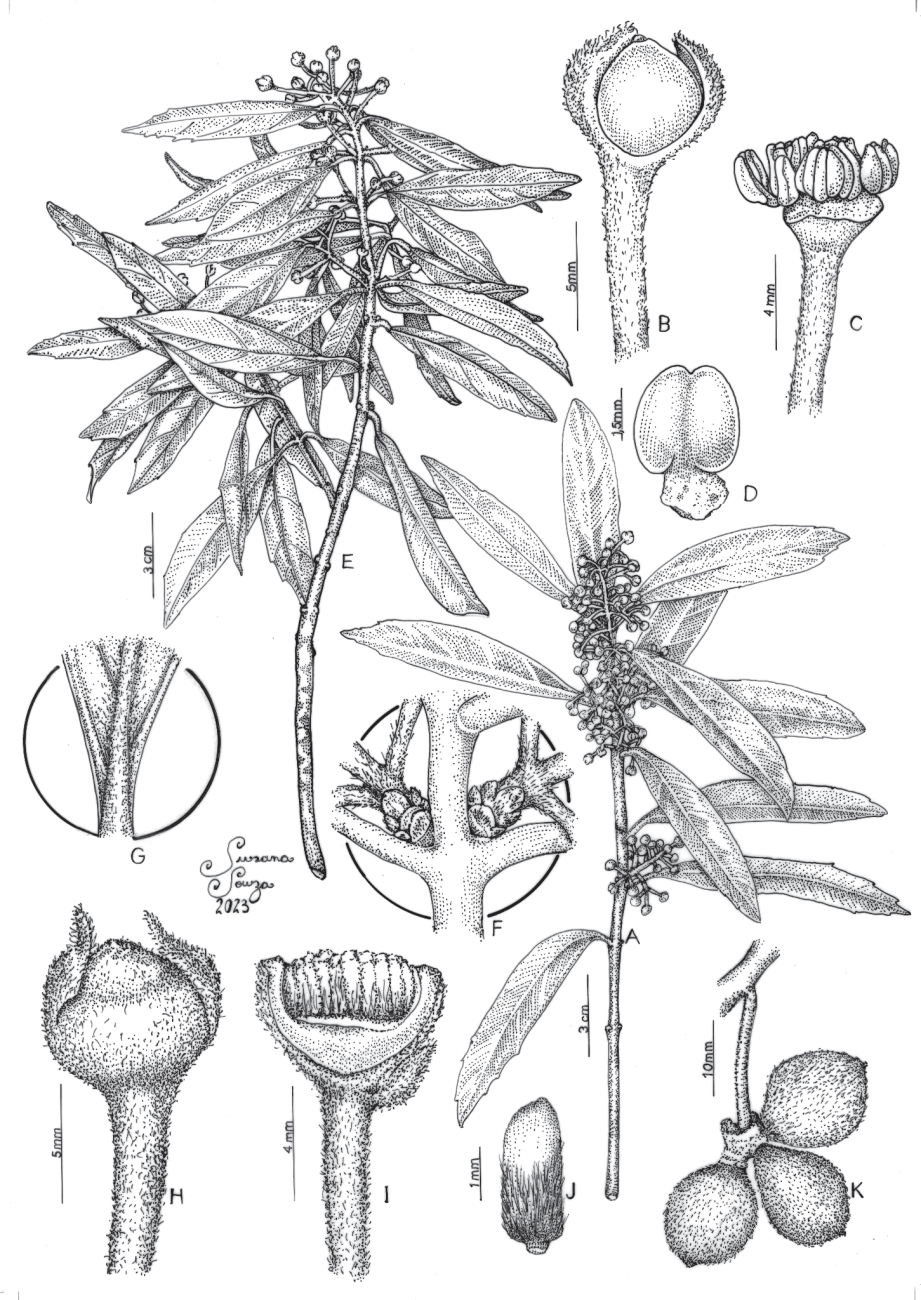
**A** branch with staminate flowers **B** staminate flower in lateral view **C** staminate flower with tepals removed, showing the stamens **D** stamen **E** branch with pistillate flowers **F** detail of cataphylls **G** detail of leaf abaxial surface **H** pistillate flower in lateral view **I** pistillate flower dissected, showing the carpels **J** carpel **K** composed fruit. (Illustrated by Susana Souza based on Zavatin 1326 and 1327 and Pedrosa 46 specimens).

**Figure 3.**
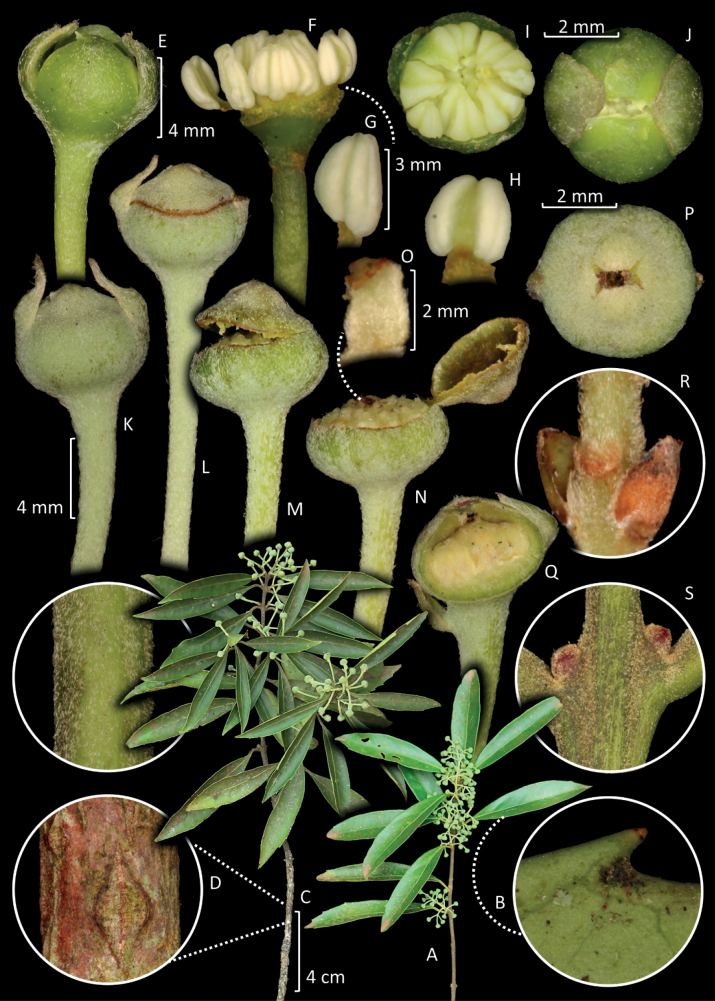
**A** branch with staminate flowers **B** monimioid tooth **C** branch with pistillate flowers **D** magnification of a young reddish branch showing a lenticel **E** staminate flower **F** staminate flower with the removal of tepals showing the androecium **G-H** stamens **I** staminate flower with removed tepals in upper view **J** staminate flower in upper view, almost complete anthesis **K** pistillate flower **L** pistillate flower starting scission in the tepals **M** early opening of calyptra **N** opened calyptra exposing the carpels **O** carpel **P** pistillate flower in upper view in anthesis **Q** pistillate flower in longitudinal section **R** bractlets **S** cataphylls. (Image authorship: Danilo A. Zavatin).

#### Phenology.

The species was collected with flowers in November and fruits in March.

#### Etymology.

The epithet of this species is a homage to Dra. Fátima Otavina de Souza Buturi, an inspiring Brazilian botanist who dedicates her career studying Asteraceae, mentoring several biologists and botanists, including the first author of this paper (DAZ).

#### Habitat and distribution.

**Figure 4.**
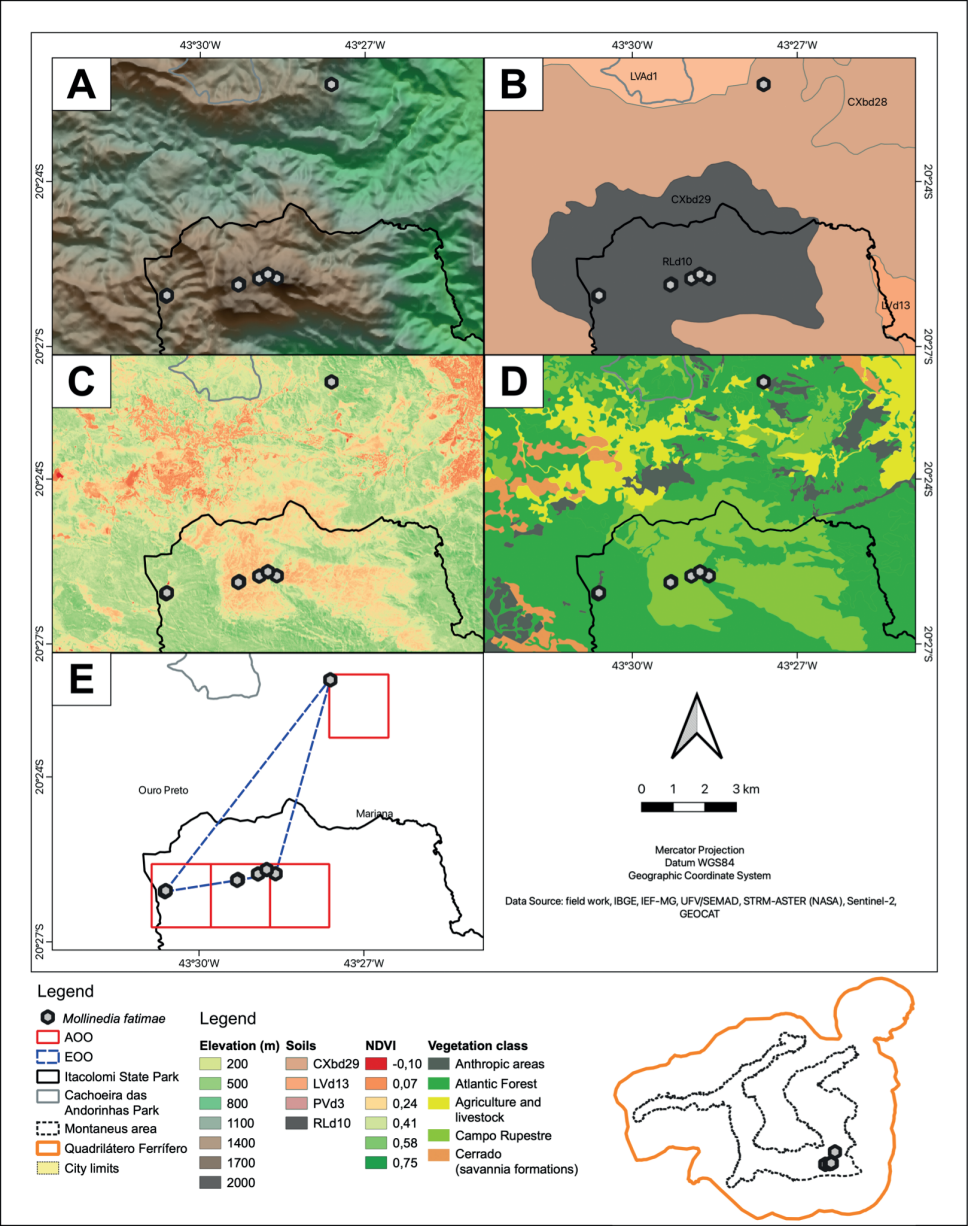
Environmental and Conservation spatial data **A** digital elevation model of the terrain **B** soil classes **C** normalized difference vegetation index (NDVI) **D** natural vegetation and anthropic use classes **E** AOO and EOO analyses.

*Mollinediafatimae* occurs at an average altitude of 1527 m (min.: 1354 m; max.: 1673 m), on mountain slopes or in river drainage ravines (Fig. 4A), predominantly in Haplic Cambisols – typical dystrophic, texture medium (code RLd10 in Fig. 4B), formed from matrix rocks of phyllite, schist, dolomite and quartzite. The vegetation is defined as mountainous semideciduous seasonal forest in intermediate to advanced stages of regeneration, with perceptible variations in the NDVI (Fig. 4C). Its individuals grow in extensive forest fragments at lower elevations but with most occurrences in small forest enclaves in areas with a predominance of “campo rupestre”, where vegetation indices indicate open formations in the surroundings (Fig. 4D).

#### Conservation status.

Most species’ populations are inside the integrally protected Itacolomi State Park, on the boundaries of the Ouro Preto and Mariana municipalities, with an EOO of 11.06 km^2^ and AOO of 16 km^2^ (Fig. 4E). According to the B1ab (iii, iv) criteria (IUCN 2012, 2022), the species should be considered critically endangered (CR). The main threats are stochastic events due to seasonal variations and climate change but mainly due to fires in the region. In high areas, fire is ignited by rare natural phenomena (i.e., lightning) and mainly by artificial fires (Schumacher et al. 2022). These fires spread quickly through campo rupestre, invading forests with significant dry biomass accumulation. Four of the six species records occur in these vegetation contact zones, with severe population decline projections if fires intensify in the region. The expansion of burned areas and the calorific power of fires have intensified in the last five years (INPE 2023). Furthermore, restricted endemism impacts potentialize the overall occurrence of the species, even with localized events.

#### Additional specimens examined.

Brazil. Minas Gerais: Ouro Preto, Parque Estadual do Itacolomi, mata em frente à capela, 20°26'04"S, 43°30'37"W, 1343 m elev., 28 Nov 2022, fem. fl., *D.A. Zavatin & L.G. Pedrosa 1326* (ALCB!, B!, BHCB!, BHZB!, DIAM!, HUEFS!, HUFU!, HURB!, INPA!, K!, MEXU!, MO!, NY!, OUPR!, SPF!, P!, PTGB!, R!, RB!, UB!, UC!, UEC!); Cratera no topo da montanha à esquerda antes do Pico do Itacolomi, 20°25'46.2"S, 43°28'56.1"W, 1594 m elev., 28 Nov 2022, mal. fl., *D.A. Zavatin & L.G. Pedrosa 1334* (BHCB!, MEXU!, OUPR!, P!, RB!, SPF!); Cratera em frente ao Pico do Itacolomi, 20°25'41.2"S, 43°28'46.1"W, 1588 m elev., 7 Nov 2022, mal. fl., *D.A. Zavatin & L.J. Sauthier 1284* (BHCB!, MEXU!, OUPR!, RB!, SPF!) Cratera no topo da montanha à esquerda antes do Pico do Itacolomi, 20°25'52.4"S, 43°29'18.1"W, 1590 m elev., 8 Nov 2022, mal. fl., *D.A. Zavatin & L.J. Sauthier 1285* (BHCB!, MEXU!, P!, RB!, SPF!); Cratera no topo da montanha à esquerda antes do Pico do Itacolomi, 20°25'52.2"S, 43°29'17.4"W, 1590 m elev., 8 Nov 2022, mal. fl., *D.A. Zavatin & L.J. Sauthier 1286* (BHCB!, MEXU!, NY!, OUPR!, P!, RB!, SPF!); Cratera no topo da montanha à esquerda do Pico do Itacolomi, 20°25'43.7"S, 43°28'36.0"W, 1596 m elev., 8 Nov 2022, mal. fl., *D.A. Zavatin & L.J. Sauthier 1287* (B!, BHCB!, HURB!, K!, MEXU!, NY!, OUPR!, P!, PTGB!, RB!, SPF!); Fazenda do Manso, 20 Mar 2018, fr., *L.G. Pedrosa 46* (OUPR!); Serra do Espinhaço, Ouro Preto, disturbed vegetation on a S-SE exposed place in the area of “Campo Grande” along the road to Cachoeira das Andorinhas, 1470–1500 m elev., 17 Sep 1990, ste., *G.L. Esteves, W. Morawetz, B. Wallnofer & J.L. da Silva 15456* (NY, W).

#### Similar species and remarks.

*Mollinediafatimae* does not co-occur with other species of the genus. The new species resembles *Mollinediaboracensis* Peixoto due to its coriaceous leaves; however, it can be easily differentiated by the length of the petioles and the indumentum of leaves and flowers. Additionally, the farinose-pilose flower indumentum in *Mollinediafatimae* resembles that in *Mollinediaarianeae* Lírio & M. Pignal and *Mollinedialeucantha* M. Molz & D. Silveira, but the species can be differentiated from *M.arianeae* by the branch indumentum, leaf consistency, and color when dried and can be distinguished from *M.leucantha* mainly by its leaf consistency, indumentum, color when dried and number of carpels. The new species was recorded in herbaria as *Mollinediaengleriana* Perkins, probably due to the dark-brown color of the leaves when dried (darkish in *M.engleriana*), but it can be differentiated from this species mainly by its leaves and flower indumentum and the length proportion of the staminate tepals in relation to the receptacle. All the comparisons with related species are summarized in Table 1.

**Table 1.** Comparison of *Mollinediafatimae sp. nov*. with the similar species *Mollinediaboracensis* Peixoto and *Mollinediaengleriana* Perkins. Characters based on additional specimens examined and Peixoto (2002), Lírio and Peixoto (2017), Molz and Silveira (2021), and Lírio et al. (2020a) and (2023b).

Characters	*Mollinediafatimae*	*Mollinediaarianeae*	*Mollinediaengleriana*	*Mollinedialeucantha*	*Mollinediaboracensis*
branch indumentum	glabrescent	tomentose	glabrous	glabrescent	glabrescent
leaf consistency	coriaceous	chartaceous	chartaceous or coriaceous	chartaceous	coriaceous
leaf width	1.5−2.8 cm	1.8−4 cm	2−10 cm	1.8−5.4 cm	3−5.5 cm
leaf color when dried	dark brown, lighter on the abaxial surface	olivaceous	Black	green-brownish, grayish brown on the abaxial surface	dark brown, lighter on the abaxial surface
leaf indumentum below	pubescent	tomentose	glabrous	tomentose	glabrous
flower indumentum	farinose and pilose	farinose and pilose	pubescent or glabrous	farinose and pilose	puberulous
tepal length in relation to flower length	tepals c. 3/4 of the flower length	tepals c. 3/4 of the flower length	tepals c. 1/2 of the flower length	tepals c. 1/4 of the flower length	tepals c. 1/2 of the flower length
number of stamens	14−20	16−21(−24)	10−20	19−20	22−24
number of carpels	12−20	unknown	12−23	22−34	26−30
geographic distribution	endemic to the Quadrilátero Ferrífero, state of Minas Gerais	endemic to Itatiaia National Park, state of Rio de Janeiro	states of Espírito Santo, Rio de Janeiro and São Paulo	states of Rio Grande do Sul and Santa Catarina	states of Rio de Janeiro and São Paulo

#### Supplementary material examined.

*Macropeplusligustrinus*. BRAZIL. Minas Gerais: Santa Bárbara, RPPN Santuário do Caraça, Trilha após a capela do Sagrado Coração, 4 Nov 2023, mal. fl., *D.A. Zavatin, R. Ramos & L.J. Sauthier* 1244 (SPF!). Belo Horizonte, Parque da Serra do Curral, Interior de mata sentido escritório da Vale, 22 May 2023, fr., *D.A. Zavatin 1765* (SPF!). Distrito Federal: Brasília, Jardim Botânico de Brasília, 28 Sep. 2021, fem. fl., *D.A. Zavatin 362* (SPF!). *Macropeplusschwackeanus*. Minas Gerais: Santa Bárbara, RPPN Santuário do Caraça, 3 Nov. 2022, mal. fl., *D.A. Zavatin, R.Ramos & L.J. Sauthier 1214* (SPF!). RPPN Santuário do Caraça, 3 Nov. 2022, fem. fl., *D.A. Zavatin, R. Ramos & L.J. Sauthier 1215* (SPF!). Monte Azul, Pico da Formosa, 28 Apr. 2023, fr., *D.A. Zavatin, F.R. de Souza & J.C.B. dos Anjos 1700* (SPF!). *Mollinediaargyrogyna*. Minas Gerais: Ouro Preto, Camarinhas, 30 Jul. 1979, mal. fl., *J. Badini s.n* (OUPR15501!). Ouro Preto, Camarinhas, 12 Sep. 1984, fem. fl., *J. Badini s.n.* (OUPR15500!). Santa Bárbara, RPPN Santuário do Caraça, 3 Nov. 2022, fr., *D.A. Zavatin, R. Ramos & L.J. Sauthier 1216*. (SPF!) *Mollinediaarianeae*. Rio de Janeiro: Serra da Mantiqueira, Maciço do Itatiaia, Parque Nacional do Itatiaia, Mata secundária entre a cascata de Maromba e cascata Véu de Noiva, 13 Aug. 1978, mal. fl., *G. Gottsberger & W. Morawetz 15-13878* (GH!, LZ!, MA!, MO!, NY!, P!, W!). Parque Nacional do Itatiaia, borda de mata rente a trilha sentido cachoeira Véu da Noiva, 6 Jun. 2022, mal. fl., *D.A. Zavatin & C. Gentile 780* (P!, NY!, SPF!). Interior de mata rente à trilha, sentido cachoeira Itaporani, 7 Jun. 2022, mal. fl., *D.A. Zavatin & C. Gentile 781* (P!, NY!, SPF!). *Mollinediaboracensis*. Rio de Janeiro: Petrópolis, Floresta IBDF, Estrada Floresta Inglesa-Pati do Alferes, 23 Apr. 1980 fr., *G. Martinelli 6729* (INPA!, MBM!, MO!, NY!, RB!). São Paulo: Santo André, Alto da Serra, Estação Biológica do Alto da Serra de Paranapiacaba, 19 Oct. 1931, mal. fl., *C. Lemos s.n* (SP29813!). Bananal, Fazenda Resgate, 22 Jun. 2005, mal. fl., *J.L. Vieira 320* (RB!). *Mollinediaengleriana*. Brazil. Espírito Santo: Santa Teresa, Estação Biológica Santa Lúcia, trilha do indaiaçú, 1 Nov. 2011, mal. fl., *E. J. Lírio 47* (RB!, MBML!). Rio de Janeiro: Nova Friburgo, Reserva Ecológica Municipal de Macaé de Cima, picada para Pedra Bicuda, 25 Jun. 1989, fem. fl., *C.M.B. Correia 34* (RB!). Minas Gerais: Lima Duarte, Parque Estadual do Ibitipoca, 21 Sep. 2006, fr., *R.C. Forzza, J.C. Silva, R.A.X. Borges, M.M. Saavedra & F.M. Ferreira 4289* (RB!). *Mollinedialeucantha*. Rio Grande do Sul: Três Cachoeiras, Lado esquerdo da RS-494, 1, 480 m da BR-101, 04 Nov. 2013, fem. fl., *D. Silveira, F. Gonzatti & L. Machado 86* (ICN!, RB!, HAS!). Morrinhos do Sul, Pixirica, Rio do Mengue, 21 Apr. 2013, fr., *D. Silveira, M. Molz & B.O. Boeni 43* (ICN!, RB!). *Mollinediaoligantha*. Minas Gerais: Santa Bárbara, EPDA de Peti, 16 Apr. 2005, fr., *R.M. Ferreira & G.S. França 96* (BHCB!). Espírito Santo: Santa Teresa, Estação Ecológica Santa Lúcia, 17 Ago. 2022, mal. fl., *D.A. Zavatin, H.L. Fonseca, A. Suzuki & E.J. Lírio 936* (SPF!). *Mollinediaovata*. Minas Gerais: Coronel Pacheco, Estação Experimental de Café, 27 Feb. 1944, mal. fl., *E.P. Heringer 1324* (RB!). Brumadinho, Inhotim, 11 Feb. 2009, fr., *J.G. Oliveira & F.M. Rodrigues 431* (RB!). Rio de Janeiro: Silva Jardim, Reserva Biológica de Poço das Antas, 26 Nov. 1992, fem. fl., *H.C. de Lima 4496* (RB!). *Mollinediaschottiana*. Minas Gerais: Ouro Preto, APA Cachoeira das Andorinhas, 27. Sep. 1999, mal. fl., *M.C.T.B. Messias 234* (OUPR!). Ouro Preto, APA Cachoeira das Andorinhas, 26 Feb. 2000, fr., *A.L. Silveira 98* (OUPR!). São Paulo: Americana, Parque da Gruta Dainese, 1 Dec. 2019, fem. fl., *D.A. Zavatin & E.Z. Viana* 153 (SPF!). *Mollinediawidgrenii*. Minas Gerais: Barroso, Mata do Baú, 21 Oct. 01, mal. fl., *A.S.M. Valente, L.C.S. Assis, R.M. Castro, M.C.M. Garcia, G.E.P. Silva & R.C. Forzza 32* (CESJ! RB!). Tiradentes, Refúgio Estadual de Vida Silvestre Libélulas de São José, 12 Nov. 2022, fem. fl., *D.A. Zavatin & L.J. Sauthier 1323* (SPF!). São Paulo: Piracicaba, ESALQ-USP, Mata próxima ao herbário, 18 Apr. 2022, fr., *D.A. Zavatin 670* (SPF!).

#### Monimiaceae in the QF.

Seven species of Monimiaceae occur in the QF region, two of the genus *Macropeplus* [*Macropeplusschwackeanus* (Perkins) I.Santos & Peixoto and *Macropeplusligustrinus* (Tul.) Perkins] and four of *Mollinedia* [*Mollinediaargyrogyna* Perkins, *Mollinediaoligantha* Perkins, *Mollinediaovata* Ruiz & Pav., *Mollinediaschottiana* (Spreng.) Perkins and *Mollinediawidgrenii* A.DC.]. *Mollinediafatimae* sp. nov. is the eighth Monimiaceae species reported to occur in the QF and the only species endemic to this region.

## Supplementary Material

XML Treatment for Mollinedia fatimae

## ﻿Additional information

### Author ORCIDs

Danilo Alvarenga Zavatin https://orcid.org/0000-0002-8851-0202

Renato Ramos https://orcid.org/0000-0001-5957-3573

Mauricio Takashi Coutinho Watanabe https://orcid.org/0000-0001-9690-5565

Elton John de Lírio https://orcid.org/0000-0002-9986-9640

### Data availability

All of the data that support the findings of this study are available in the main text or Supplementary Information.
